# Supplementary material for: Resolving the full spectrum of human genome variation using Linked-Reads
Source: Genome Res. 2019 Apr;29(4):635–45. doi: 10.1101/gr.234443.118 (PMC6442396; doi:10.1101/gr.234443.118)
Supplement: Supplemental Material [file supp_gr.234443.118_Supplemental_longranger-2.2.zip › longranger-2.2.tar.bz2!/lr2.2-public/lib/stan/cmdstan/stan/doc/api/html/agrad_2rev_2matrix_2log__softmax_8hpp.html]

Stan: src/stan/agrad/rev/matrix/log\_softmax.hpp File Reference


|  |  |
| --- | --- |
|  | Stan  2.5.0  probability, sampling & optimization |


- Main Page
- Related Pages
- Namespaces
- Classes
- Files

- File List
- File Members

All Classes Namespaces Files Functions Variables Typedefs Enumerations Enumerator Friends Macros Pages

- src
- stan
- agrad
- rev
- matrix

Namespaces |
Functions

log\_softmax.hpp File Reference

`#include <cmath>`  
`#include <vector>`  
`#include <stdexcept>`  
`#include <stan/math/matrix/Eigen.hpp>`  
`#include <stan/math/matrix/log_softmax.hpp>`  
`#include <stan/math/matrix/softmax.hpp>`  
`#include <stan/agrad/rev/var.hpp>`  
`#include <stan/math/error_handling/matrix/check_nonzero_size.hpp>`

Go to the source code of this file.

|  |  |
| --- | --- |
| Namespaces | |
|  | stan |
|  | Probability, optimization and sampling library. |
|  | |
|  | stan::agrad |
|  | Function gradients via reverse-mode automatic differentiation. |
|  | |

|  |  |
| --- | --- |
| Functions | |
| Eigen::Matrix< var,   Eigen::Dynamic, 1 > | stan::agrad::log\_softmax (const Eigen::Matrix< var, Eigen::Dynamic, 1 > &alpha) |
|  | Return the softmax of the specified Eigen vector. More... |
|  | |

## Variable Documentation

vari\*\* alpha\_

Definition at line 20 of file log\_softmax.hpp.

const int idx\_

Definition at line 23 of file log\_softmax.hpp.

const int size\_

Definition at line 22 of file log\_softmax.hpp.

const double\* softmax\_alpha\_

Definition at line 21 of file log\_softmax.hpp.


---

[ Stan Home Page ]

*© 2011–2014,
Stan Development Team.*
